# A large-scale multivariate soccer athlete health, performance, and position monitoring dataset

**DOI:** 10.1038/s41597-024-03386-x

**Published:** 2024-05-30

**Authors:** Cise Midoglu, Andreas Kjæreng Winther, Matthias Boeker, Susann Dahl Pettersen, Sigurd Pedersen, Nourhan Ragab, Tomas Kupka, Steven A. Hicks, Morten Bredsgaard Randers, Ramesh Jain, Håvard J. Dagenborg, Svein Arne Pettersen, Dag Johansen, Michael A. Riegler, Pål Halvorsen

**Affiliations:** 1 SimulaMet, Oslo, Norway; 2 UiT The Arctic University of Norway, Tromsø, Norway; 3 Forzasys, Oslo, Norway; 4 University of Southern Denmark, Odense, Denmark; 5 University of California, Irvine, CA USA; 6 Oslo Metropolitan University, Oslo, Norway

**Keywords:** Medical research, Physiology

## Abstract

Data analysis for athletic performance optimization and injury prevention is of tremendous interest to sports teams and the scientific community. However, sports data are often sparse and hard to obtain due to legal restrictions, unwillingness to share, and lack of personnel resources to be assigned to the tedious process of data curation. These constraints make it difficult to develop automated systems for analysis, which require large datasets for learning. We therefore present *SoccerMon*, the largest soccer athlete dataset available today containing both subjective and objective metrics, collected from two different elite women’s soccer teams over two years. Our dataset contains 33,849 subjective reports and 10,075 objective reports, the latter including over six billion GPS position measurements. *SoccerMon* can not only play a valuable role in developing better analysis and prediction systems for soccer, but also inspire similar data collection activities in other domains which can benefit from subjective athlete reports, GPS position information, and/or time-series data in general.

## Background & Summary

A key development in sports over the last three decades has been the increased use of scientific methods to improve the preparation for and participation performance in elite competitions. In this context, international sports are undergoing a revolution fueled by the rapidly increasing availability of quantifiable athlete performance data, sensor technologies, and software for advanced analytics. These recent innovations have enabled the close monitoring of athlete performance across all training sessions and matches, facilitating a much deeper understanding of training methods that benefit athletes and coaches alike, i.e., empowering both players and involved personnel to quantify individual and team strengths and weaknesses, give feedback on physical performance, psychological status, and wellness, educate personnel, plan training content in micro- and macrocycles, and avoid overuse injuries.

In this context, soccer (also known as association football) is an open-loop sport with many parameters that can be analyzed. The performance is a multifactorial construct with a dynamic and stochastic nature, where the physical performance of players (e.g., high-speed activities) is affected by external factors (e.g., ball possession and period of the season), which consequently cause fluctuations over time^1^. Several studies have reported on the effect of variability between players, positions, and matches^2–12^. In addition, there has been an increasing focus on the value of a player’s mentality and their subjective perception of their training load and well-being^11,13^. Furthermore, little research has been done within this field in the context of women’s soccer. Although both men and women play the same game, research on other sports such as weightlifting^14^ and cycling^15^ have shown differences in training response between women and men^16^. In light of these, there is a need for more research on the effects of various mental and physical parameters in women’s soccer.

Analysis of subjective and objective data can provide vital insights for individual training personalization and injury prevention, but it might also consume a lot of manual labor. Therefore, an important goal is to have automated systems which can generate and analyze large amounts of data from training sessions and matches in professional soccer, and provide detailed insights and predictions about the future status. Such automated systems can enable individualization of training, improve player selection for upcoming matches, and avoid overuse injuries through the detection of anomalies. However, a considerable challenge to performing research in this area, whether the analysis is manual or computer-based and automated, is the availability of data. This is especially true for women’s soccer. Existing soccer datasets, such as datasets for event detection containing video and image annotations^17–21^, player tracking datasets^18,22,23^, and datasets from tournaments containing match information (https://data.world/datasets/soccer, https://datahub.io/collections/football), focus only on male players. To the best of our knowledge, there is no existing dataset for female players which includes players’ subjective assessments of their own physical and psychological parameters, as well as objective measurements of locomotor activity during training sessions and matches. Therefore, we present such a dataset containing both objective and subjective metrics for women’s soccer. Our previous work has made use of position data from GPS and radio-wave local positioning systems^9,12,24–26^, and subjective training load and wellness reports^27–29^, where we have both analyzed the gameplay and performed predictions on future performance. During this period, we observed the need for open and shareable datasets, which has become even more prominent in recent years due to the rise of machine learning applications, where reproducible and comparable results are one of the biggest challenges associated with these new algorithms^30^.

In this paper, we present the *SoccerMon* dataset, which is composed of data collected and used by professional teams in the Norwegian women’s elite soccer league Toppserien during the 2020 and 2021 seasons. In particular, teams have: (1) used the *PmSys* athlete monitoring system^28,31–33^ to collect subjective reports, which log parameters related to training load, wellness, injuries, illnesses, and game performance, and (2) used wearable GPS tracking equipment (STATSports APEX GNSS tracking system) during most of their training sessions and some matches to collect objective reports, which log parameters related to position, acceleration, rotation, and heart rate. Overall, we collected data from two elite women’s soccer teams over two years, comprising 33,849 subjective reports and 10,075 objective reports, the latter including 6,248,770,794 GPS measurements.

## Methods

The *SoccerMon* dataset has been curated using data from two systems: subjective reports collected from the *PmSys* athlete monitoring system^28^ and objective reports collected from the STATSports APEX GNSS tracking system. We collected both subjective and objective data over two years (during the 2020 and 2021 seasons) from two different teams in the Norwegian women’s elite soccer league. All players have given their written informed consent to participate, including the open publication of the recorded data. After the raw export from the two systems, all player metadata have been removed, and all files have been renamed with randomly generated strings. Apart from this anonymization, neither the subjective nor the objective data have been altered in any way. The study was approved by the Norwegian Privacy Data Protection Authority (reference number: 296155), and exempted from further user consent. The study was also exempted from approval by the Regional Committee for Medical and Health Research Ethics - South East Norway and the Regional Committee for Medical and Health Research Ethics - Northern Norway, as the collection of the data did not include a bio-bank, medical or health data related to illness, or interfere with the normal operation of the players.

### Collection of subjective reports

**Fig. 1:**
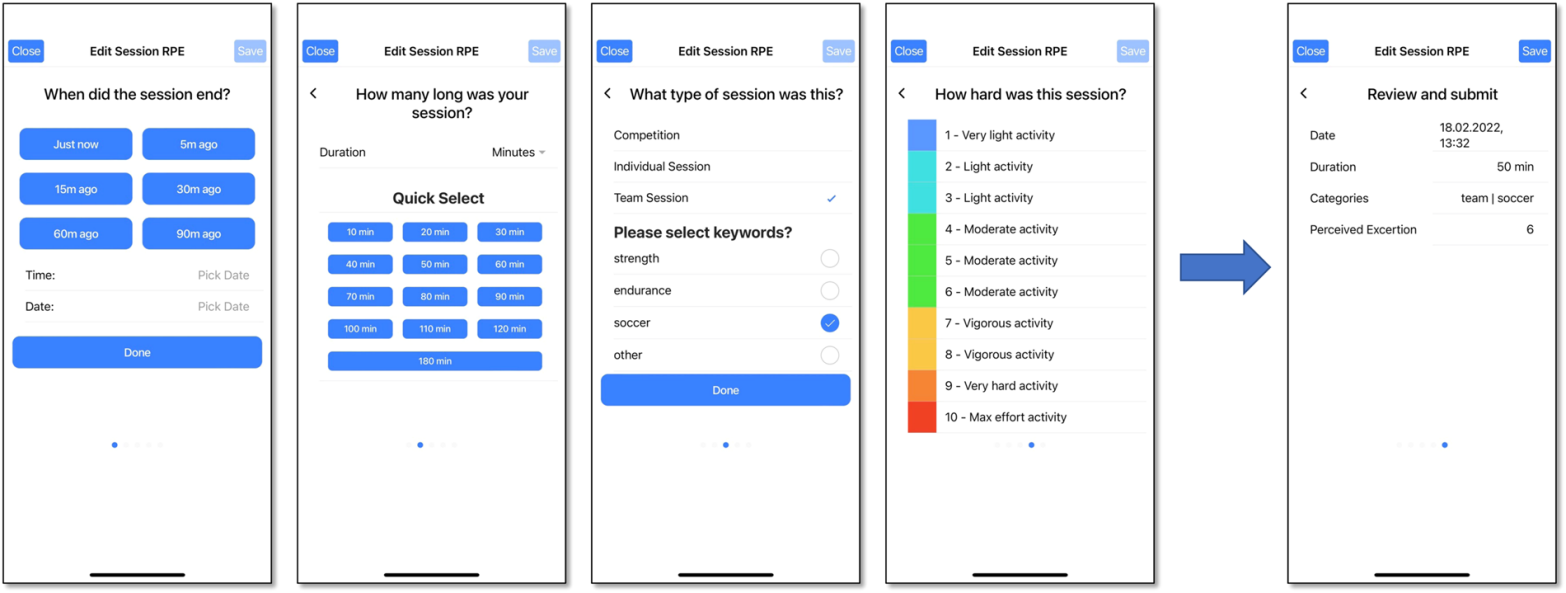
Reporting of sRPE in *PmSys*.

The first part of the *SoccerMon* dataset consists of subjective metrics related to perceived training load, wellness, injuries, illnesses, and game performance. These metrics have been reported by players using the *PmSys* athlete monitoring system^28^. The *PmSys* system was developed for controlled systematic longitudinal data collection and extraction, enabling fast and efficient monitoring of athletes’ phenotypic and self-reported metrics, based on their perceived training load, wellness, injuries, illnesses, and game performance. In particular, for reporting metrics, players interacted with the *PmSys* system using the *Reporter App* available for both Android and iOS platforms. This smartphone application allows easy access to a player profile for submitting reports, as described below. Training load reports were submitted by players per training or activity session, wellness reports per day, game performance reports per match (game), and injury and illness reports whenever these occurred. Player reports were first stored locally on their smartphone, then synchronized with the *PmSys* cloud server as soon as they had a network connection, which allowed players to complete reporting even if they were offline. In accordance with privacy-preserving guidelines, each report is owned by the player who generated it, and a player must actively attach their profile to a team for coaches and other personnel to be able to view their data through a trainer portal. The following reports were collected with the *PmSys* app:

- **Training Load Reports:** The app allows for submitting training load reports through the questionnaire depicted in Fig. 1. This questionnaire is intended for use after every training or activity session including games. The players reported the duration of the session, the type of the session, and a 1 to 10 score of how hard they perceived the session to be, i.e., their rating of perceived exertion (RPE) for the session. Table 1 lists the training load report parameters collected using the *PmSys* system. Based on these raw parameters, a wider range of training load metrics were calculated, including session RPE (sRPE), daily load, weekly load, Acute Training Load (ATL), Chronic Training Load (CTL), Acute Chronic Workload Ratio (ACWR), monotony, and strain. Table 2 lists the calculated training load metrics.

**Table 1:** Overview of subjective report parameters collected using the *PmSys* system.

Report	Granularity	Parameter	Description
Training Load Report	Per training or activity session	Timestamp	Date and time for session
		Type of session	Competition or individual session or team session, along with keywords
		Session duration	Session duration in hours and/or minutes
		RPE	Rate of perceived exertion (1–10)
Wellness Report	Per day	Timestamp	Date for which the report applies
		Fatigue	Fatigue (1–5)
		Mood	Mood (1–5)
		Readiness	Readiness to train (1–10)
		Sleep duration	Sleep duration for the previous night in hours and/or minutes
		Sleep quality	Sleep quality for the previous night (1–5)
		Soreness	Muscle soreness (1–5)
		Stress	Stress (1–10)
Injury Report	Per injury	Timestamp	Date and time for injury
		Location	Area of injury marked on body silhouette
		Severity	Severity of injury indicated as either minor or major
Illness Report	Per illness	Timestamp	Date and time for illness
		Symptoms	Illness symptoms selected among list
Game Performance Report	Per game	Timestamp	Date and time for game
		Team performance	Overall team performance (1–10)
		Defensive performance	Individual defensive performance (1–10)
		Offensive performance	Individual offensive performance (1–10)

**Table 2:** Overview of additional training load metrics calculated from the raw parameters (session duration and RPE) in the training load reports.

Metric	Description	Formula
Session RPE (sRPE)	The workload for a single session based on the session duration and the reported RPE	*RPE*·*duration*
(Daily) Training Load	The sum of sRPE for a day	$\sum sRPE$ per day
Weekly Load (WL)	The sum of sRPE over the last 7 days	$\sum sRPE$ per week
Acute Training Load (ATL)	The current level of fatigue (average sRPE over the last 7 days)	${7}^{-1}{\sum }_{n=i}^{i+7}D{L}_{i}$
Chronic Training Load (CTL)	The cumulative training dose that builds up over a longer period of time (average sRPE over the last 28 or 42 days)	${x}^{-1}{\sum }_{n=i}^{i+x}D{L}_{i},\;\;{\rm{x}}=28\;{\rm{or}}\;42$
Acute Chronic Workload Ratio (ACWR)	An indication of whether an athlete is in a well-prepared state, or at an increased risk of getting injured (ATL divided by CTL)	*ATL*·*CTL*^−1^
Monotony	Training variation across the last 7 days (average sRPE over the last 7 days divided by the standard deviation (SD))	*ATL*·*SD*^−1^
Strain	Overall training stress from the last 7 days (total weekly sRPE multiplied with Monotony)	*WL*·*Monotony*
- **Wellness Reports:** The app has a questionnaire for reporting wellness, as depicted in Fig. 2. This questionnaire is intended for daily use, i.e., each player was instructed to report wellness once a day. Wellness parameters include fatigue, mood, readiness to train, sleep duration, sleep quality, muscle soreness, stress, and optionally menstruation. Readiness is reported on a scale from 1 to 10, sleep duration in hours and minutes, and menstruation with a checkbox. The remaining metrics are reported on a scale from 1 to 5. All scales in the questionnaire have supporting textual explanations for a more consistent interpretation of the numbers. Table 1 lists the wellness report parameters collected using the *PmSys* system.

**Fig. 2:**
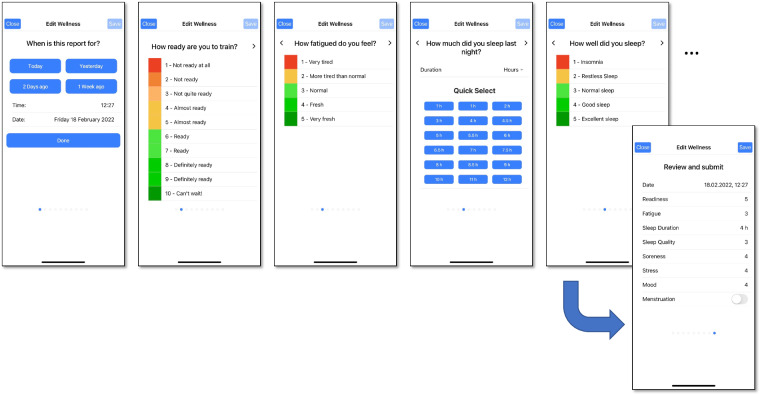
Reporting of wellness in *PmSys*.
- **Injury Reports:** Injuries can be reported using the app. Injury reports constitute a subjective notification of injury location and severity. No diagnoses are included, as these are meant to be made by medical personnel. The reporting procedure is similar to previous examples, with the corresponding questionnaire illustrated in Fig. 3. The questionnaire displays a body silhouette on which the players can tap to mark injury locations (from among 22 location alternatives): single tap means minor pain (marked as orange) and double tap means major pain (marked as red). Players can submit as many injury reports as needed, without any requirements on reporting frequency, and each report can include as many injuries as the player wishes to report (with a maximum of 22, indicating injuries in all possible body locations). All injury reports are recorded separately, and the injuries listed in reports from consecutive days are not merged. Table 1 lists the injury report parameters collected using the *PmSys* system.

**Fig. 3:**
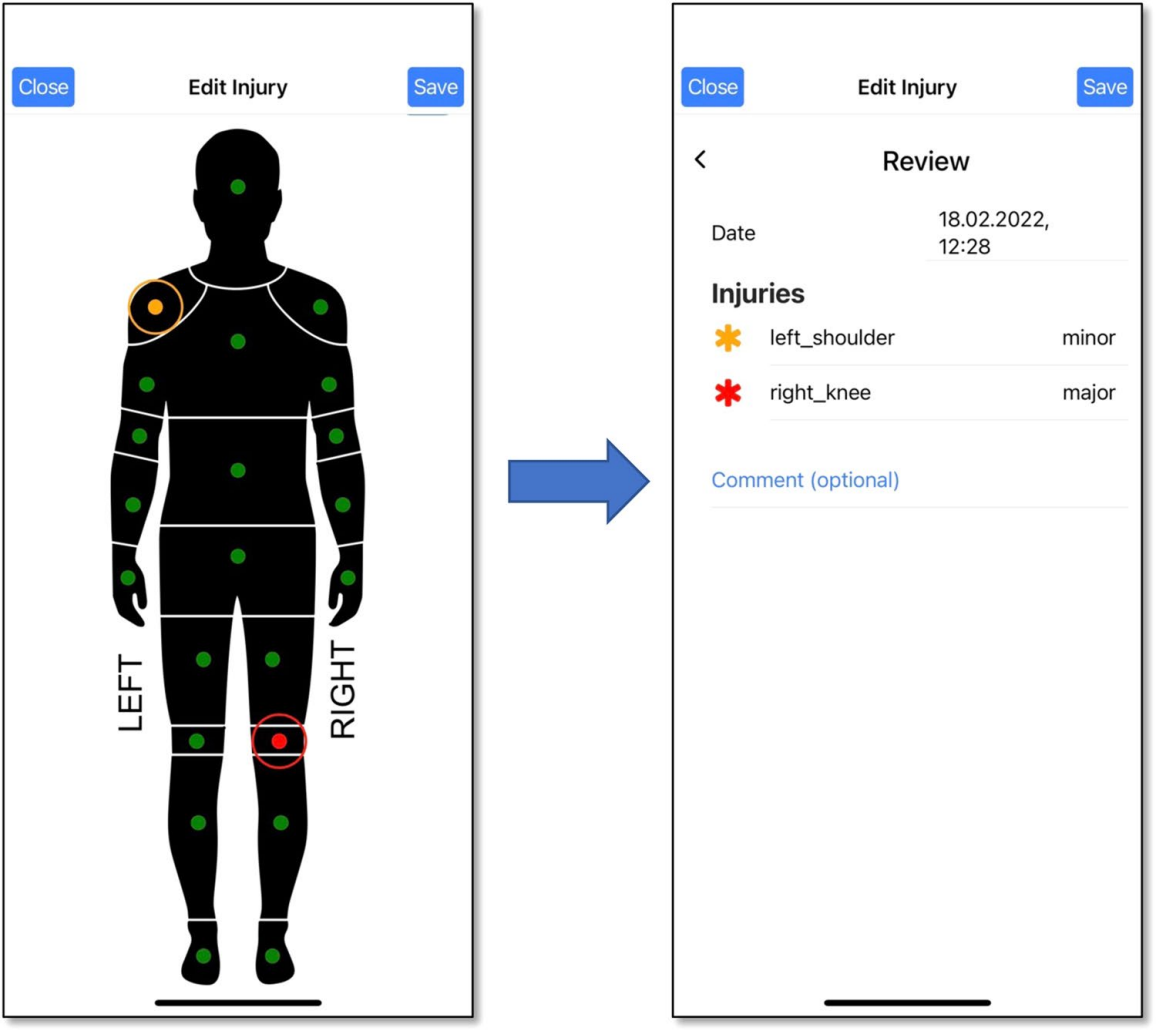
Reporting of injuries in *PmSys*.
- **Illness Reports:** Using the app, illnesses can be reported via selection from a list of symptoms, as illustrated in Fig. 4. Multiple illness symptoms can be reported in the same illness report. Similar to injuries, this notification only constitutes a subjective notification of perceived symptoms, and is not validated by medical personnel. Table 1 lists the illness report parameters collected using the *PmSys* system.

**Fig. 4:**
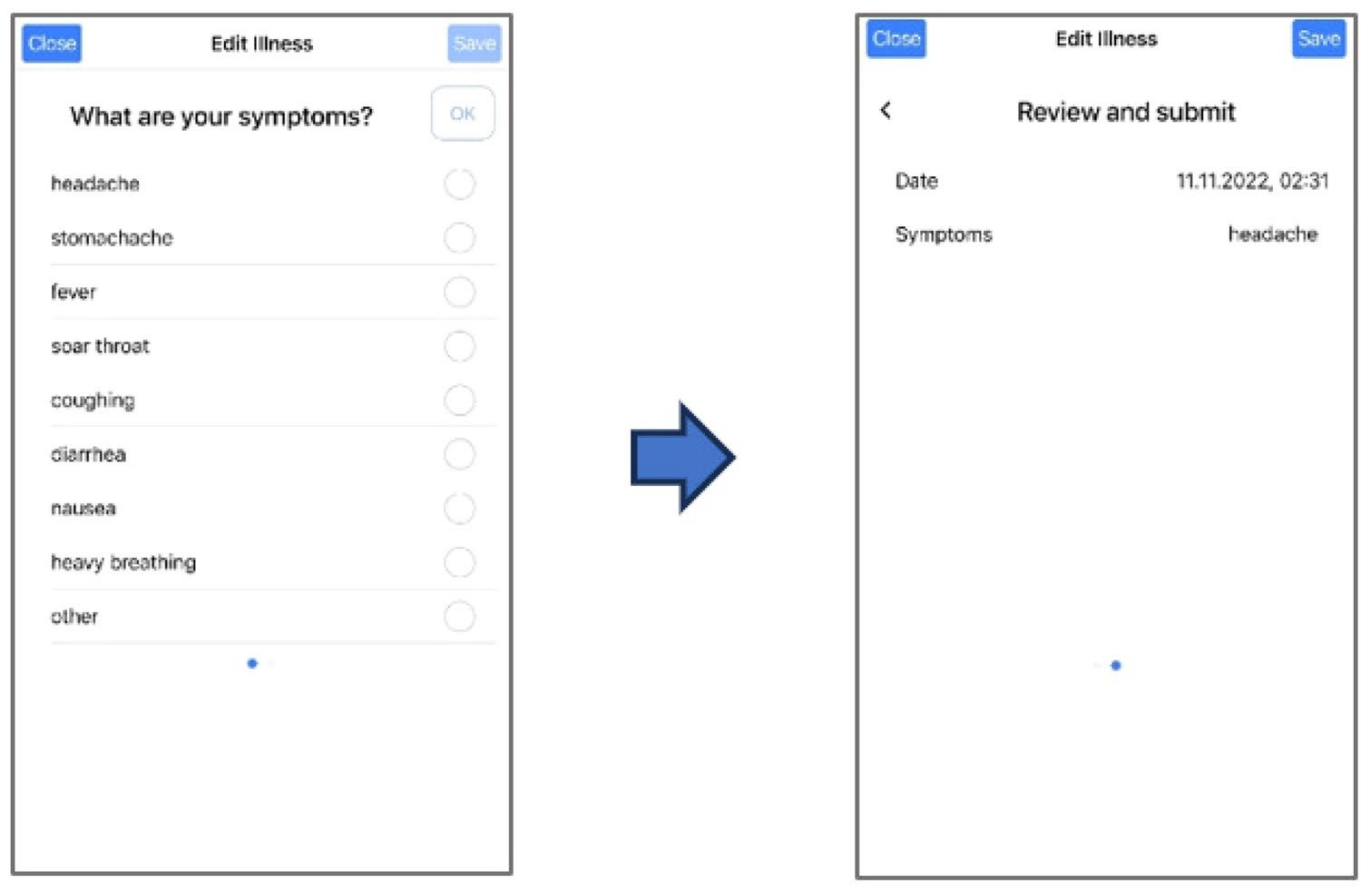
Reporting of illnesses in *PmSys*.
- **Game Performance Reports:** The app allows for submitting game reports through the questionnaire depicted in Fig. 5. This questionnaire is intended for use after every game. The players reported the time of the game, their rating of the overall team performance, their rating of their individual defensive performance, and their rating of their individual offensive performance, on a scale from 1 to 10. Table 1 lists the game performance report parameters collected using the *PmSys* system.

**Fig. 5:**
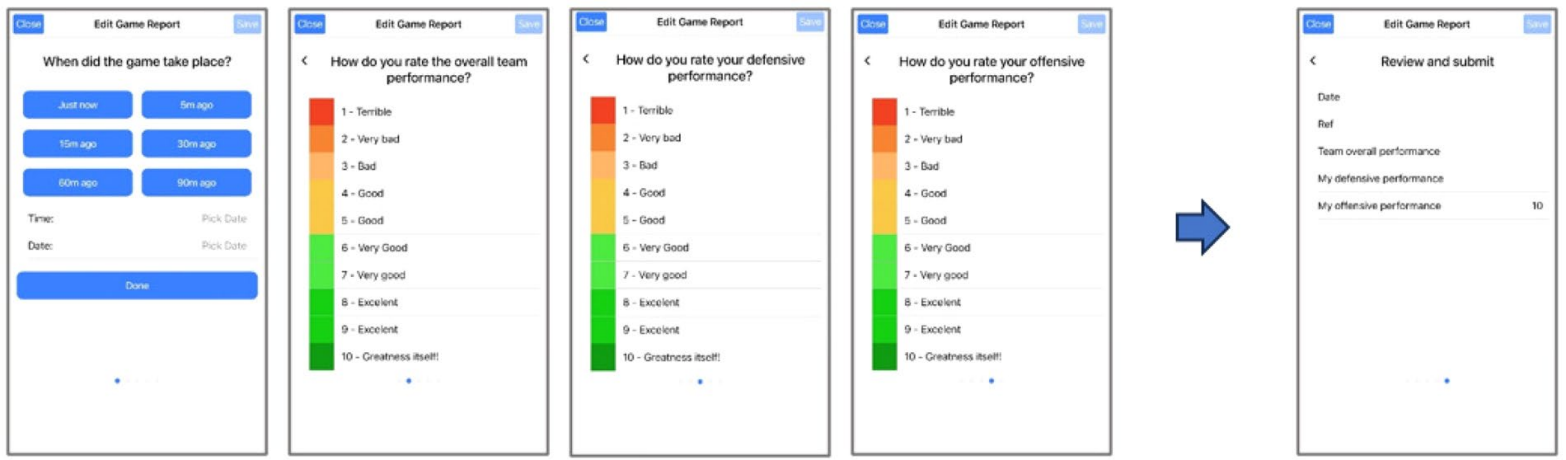
Reporting of game performance in *PmSys*.

For the curation of the *SoccerMon* dataset, these reports were exported in full and tagged with a player identifier. Player identifiers were only used to match the subjective reports to the objective reports described below, and after the matching, all explicit identifiers were replaced with random anonymized player IDs which are common for both the subjective and objective reports. Apart from the anonymization, the subjective data from the *PmSys* athlete monitoring system have not been altered in any way.

### Collection of objective reports

**Fig. 6:**
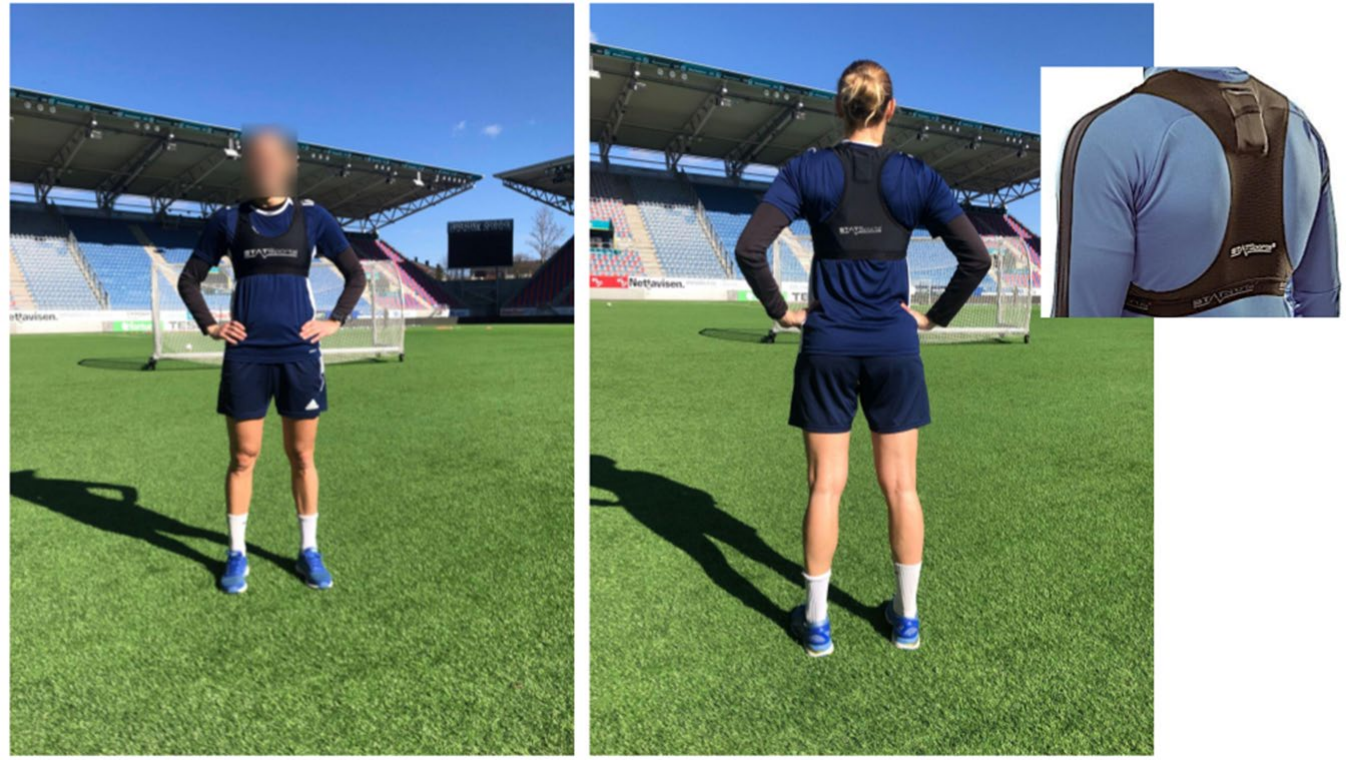
STATSports GPS tracker in vest during training.

The second part of the *SoccerMon* dataset consists of objective metrics related to position and movement on the soccer pitch, acceleration, rotation, and heart rate. These metrics have been collected by the FIFA-approved STATSports APEX GNSS tracking system. Players were equipped with the tracking system, which contains 10 Hz multi-GNSS augmented units capable of concurrently tracking multiple satellite systems (e.g., global positioning system (GPS), GLONASS, Galileo and BeiDou), a tri-axial accelerometer (952 Hz), a magnetometer (10 Hz), and a tri-axial gyroscope (952 Hz), embedded in the STATSports vest shown in Fig. 6.

- **GPS Reports:** During both training sessions and competitions, each player wore a sensor unit, roughly the size of a matchbox, on their upper back in the tight-fitted vest. Players also had the option of wearing a chest strap to monitor heart rate. To minimize inter-device error, each player used the same sensor unit during the entire season. The measurement data from the sensor units were retrieved via the manufacturer’s software (STATSports Sonra 2.1.4) after each session. Consequently, from each player, one GPS report was collected for each training or activity session during which they wore their sensor unit. Table 3 provides an overview of the GPS report parameters collected using the STATSports APEX GNSS tracking system.

**Table 3:** Overview of objective report parameters collected using the STATSports system.

Sensor	Parameter	Description	Frequency
NA	time	Timestamp for record with millisecond granularity (HH:mm:ss.SSS)	NA
	id	Sensor unit ID (replaced with anonymized athlete ID)	NA
Heart rate monitor	heart_rate	Heart rate in beats per minute (*b*·*m*^−1^)	10 Hz
GPS	lat	Latitude in degrees (°)	10 Hz
	lon	Longitude in degrees (°)	10 Hz
	speed	Sensor-calculated speed in meters per second (*m*·*s*^−1^)	10 Hz
	hacc	Horizontal accuracy	10 Hz
	hdop	Horizontal dilution of precision	10 Hz
	signal_quality	Quality of the GNSS/GPS signal	10 Hz
	num_satellites	Total number of satellites used in the calculation of the unit’s position	10 Hz
Accelerometer	inst_acc_impulse	Absolute acceleration in meters per second per second (*m*·*s*^−2^)	10 Hz
	accl_x	Acceleration along x-axis (*g*)	100 Hz
	accl_y	Acceleration along y-axis (*g*)	100 Hz
	accl_z	Acceleration along z-axis (*g*)	100 Hz
Gyroscope	gyro_x	Rate of rotation along x-axis in degrees per second (*deg*·*s*^−1^)	100 Hz
	gyro_y	Rate of rotation along y-axis in degrees per second (*deg*·*s*^−1^)	100 Hz
	gyro_z	Rate of rotation along z-axis in degrees per second (*deg*·*s*^−1^)	100 Hz

For the curation of the *SoccerMon* dataset, these reports were exported in full and tagged with a player identifier. It should be mentioned that the raw data is likely to have been pre-filtered by the receiver’s firmware to reduce the noise from the satellite systems^34^. Player identifiers were only used to match the objective reports to the subjective reports described above, and after the matching, all explicit identifiers were replaced with random anonymized player IDs which are common for both the subjective and objective reports. In addition to the anonymization, column names in each file were cleaned up, and the raw data were converted from the original CSV format to *parquet* format for compression purposes (see further comments in the Usage Notes section). Apart from these changes, the objective data from the STATSports APEX GNSS tracking system have not been altered in any way.

## Data Records

The *SoccerMon* dataset is available from Zenodo^35^. *SoccerMon* is open access and licensed under Creative Commons Attribution 4.0 International (CC BY 4.0). The dataset includes a total of 33,849 subjective reports and 10,075 objective reports. The data records have a total size of around 93GB, where subjective reports make up 9.3MB and objective reports make up 92.3GB.

### Subjective metrics

As shown in the overview Table 4, the *SoccerMon* dataset contains 33,849 subjective reports in total, which are made up of 16,261 training load reports, 17,002 wellness reports, 306 injury reports, 32 illness reports, and 248 game performance reports. Subjective reports were collected from a total of 50 players, with 27 players from Team A and 23 players from Team B.

**Fig. 7:**
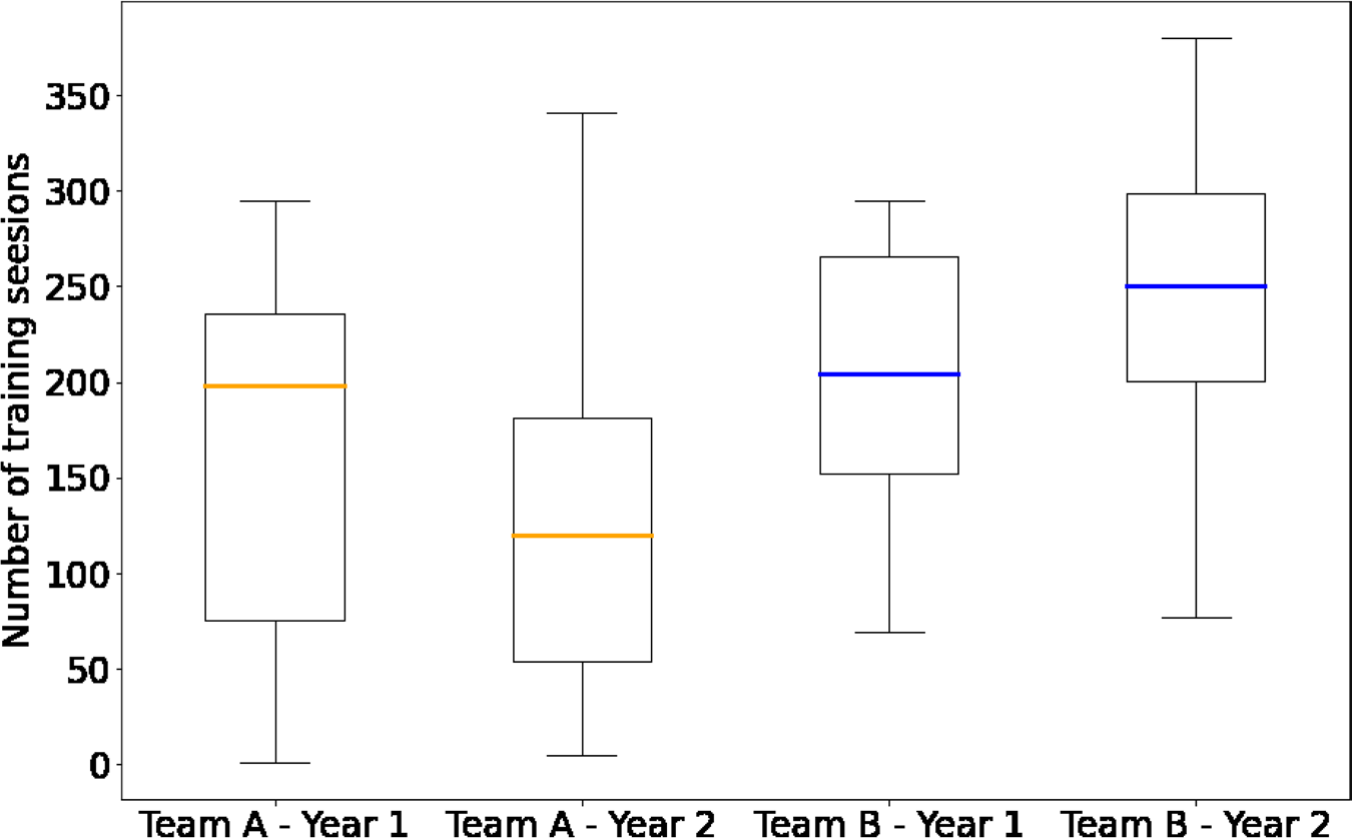
Distribution of the number of training sessions per player, presented as boxplots for each team and year. Each boxplot summarizes the probability distribution, with the lower and upper box bounds representing the first and third quartile, respectively. The middle line displays the median. The two lines outside of the box show the minimum and maximum of the distribution. Points present outliers.
- **Wellness metrics:** As described in the previous section, players were instructed to submit wellness reports daily. However, the self-reported wellness parameters reveal a fair amount of missing data (days for which no wellness report was submitted by a given player). Overall, 17,002 reports were retrieved in total, with 11,026 reports from Team A and 5,976 reports from Team B. In the *SoccerMon* dataset, 7 wellness metrics are available, as listed in Table 1.
- **Injury metrics:** A total of 162 injury reports including 306 injuries were submitted from both teams over two years. Figure 8 provides an overview of the location and severity of all injuries reported by both teams. Most severe injuries were reported in the knees, especially the right knee. The number of minor injuries is fairly evenly distributed over the lower body parts. In the *SoccerMon* dataset, 2 injury metrics are available, as listed in Table 1.

- **Training load metrics:** As described in the previous section, training load reports were collected per training or activity session. Overall, Team A logged 11,052 sessions, and Team B logged 5,209 sessions (making up a total of 16,261 reports). Each player completed a number of training sessions, comprising both team and individual sessions. Thus, the number of sessions per player is not fixed, but a distribution depending on team, player, and year. The distributions are presented as boxplots in Fig. 7. Team A has a lower annual median of 374 and 382 reported sessions per player, while Team B has an annual median of 389 and 390 sessions per player, in 2020 and 2021 respectively. In the *SoccerMon* dataset, 11 training load metrics are available, including both the raw parameters listed in Table 1, and the calculated metrics listed in Table 2.

**Fig. 8:**
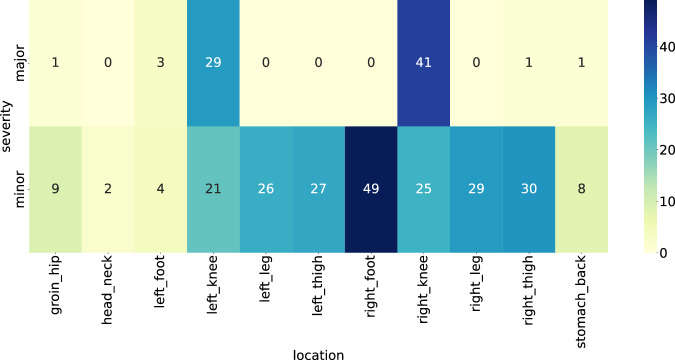
Overview of the location and severity of injuries reported by both teams over two years.
- **Illness metrics:** A total of 15 illness reports including 32 illness symptoms were submitted from both teams over two years. In the *SoccerMon* dataset, 1 illness metric is available, as listed in Table 1.
- **Game performance metrics:** As described in the previous section, game performance reports were collected per match (game). A total of 248 game performance reports were submitted from both teams over two years. In the *SoccerMon* dataset, 3 game performance metrics are available, as listed in Table 1.

**Table 4:** Overview of all data records in the *SoccerMon* dataset, in terms of the number of metrics and samples per report type.

Part	Report Type	Num. Metrics	Number of Samples		
			Total	Team A	Team B
Subjective	Training Load	11	16,261	11,052	5,209
	Wellness	7	17,002	11,026	5,976
	Injury	2	306	291	15
	Illness	1	32	17	15
	Game Performance	3	248	161	87
	TOTAL	24	33,849 (50 players)	22,547 (27 players)	11,302 (23 players)
Objective	GPS	17	10,075	5,438	4,637
	TOTAL	17	10,075 (76 players)	5,438 (39 players)	4,637 (37 players)

For training load, wellness, and game performance, the number of samples refer to the number of reports. For injury and illness, the number of samples refers to the number of injuries and illness symptoms (instead of the number of reports, as a single report might include multiple injuries or illness symptoms).

**Fig. 9:**
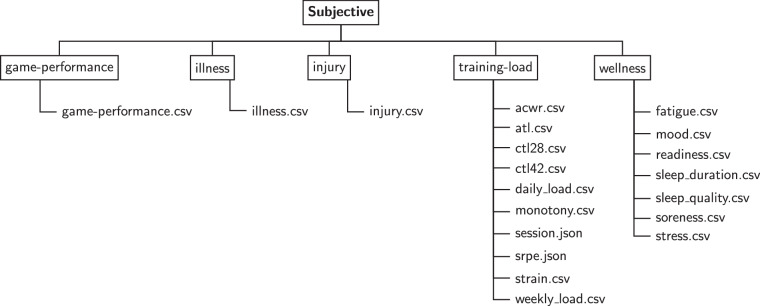
Folder structure for the first part of the *SoccerMon* dataset (subjective).

Figure 9 illustrates the folder structure for the first part of the *SoccerMon* dataset. Subjective reports are organized into folders per report type, which contain roughly one file per metric, each aggregating the data from all players over two years for the particular metric. Metrics with a per day granularity were presented in tabular form and stored in.csv files, whereas metrics with a per session granularity (which can have a different length for each player, and for which a tabular presentation is not possible) were stored in.json files.

### Objective metrics

**Fig. 10:**
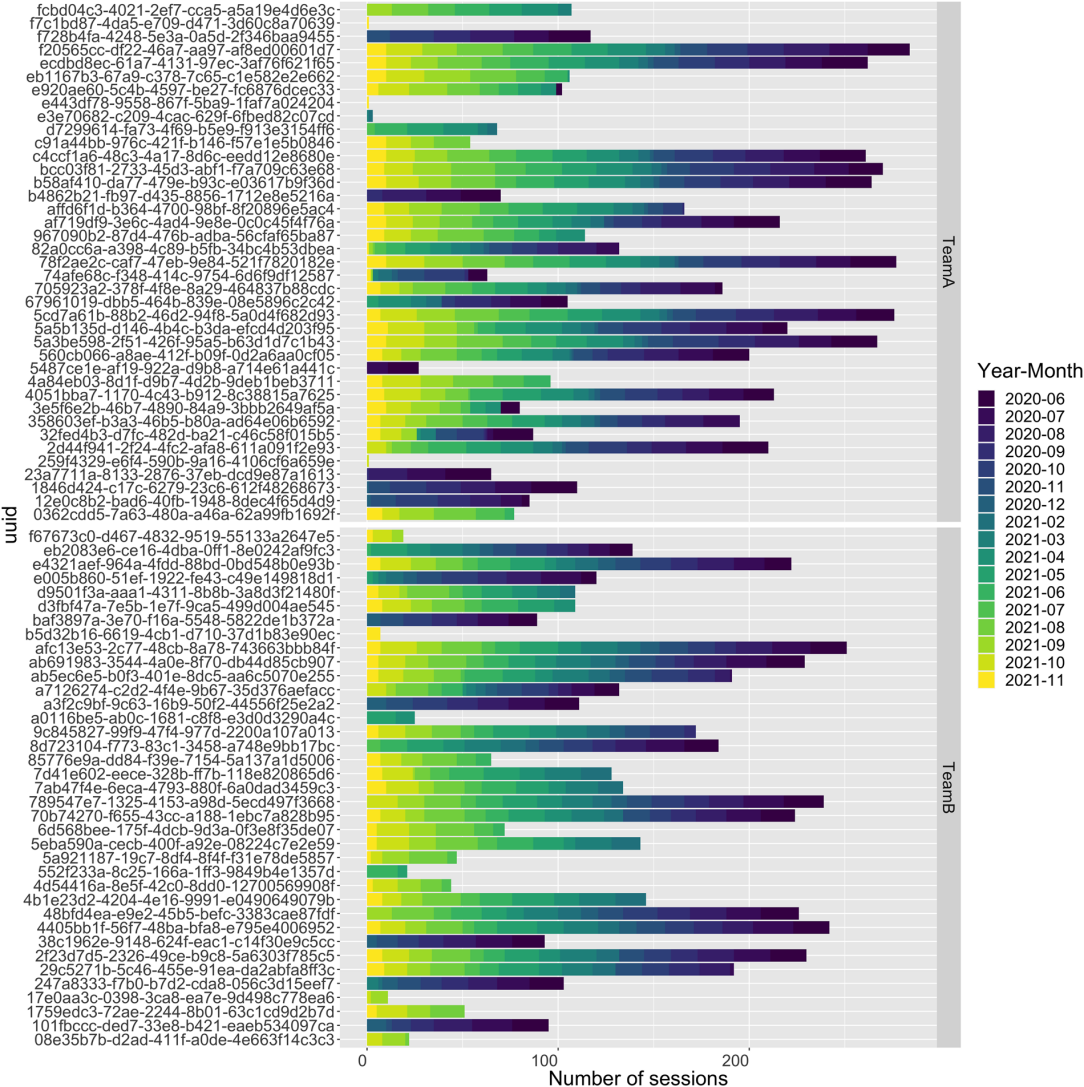
Number of GPS sessions per player, presented as a bar chart indicating anonymized player UUID, year, and month (top: Team A, bottom: Team B).

**Fig. 11:**
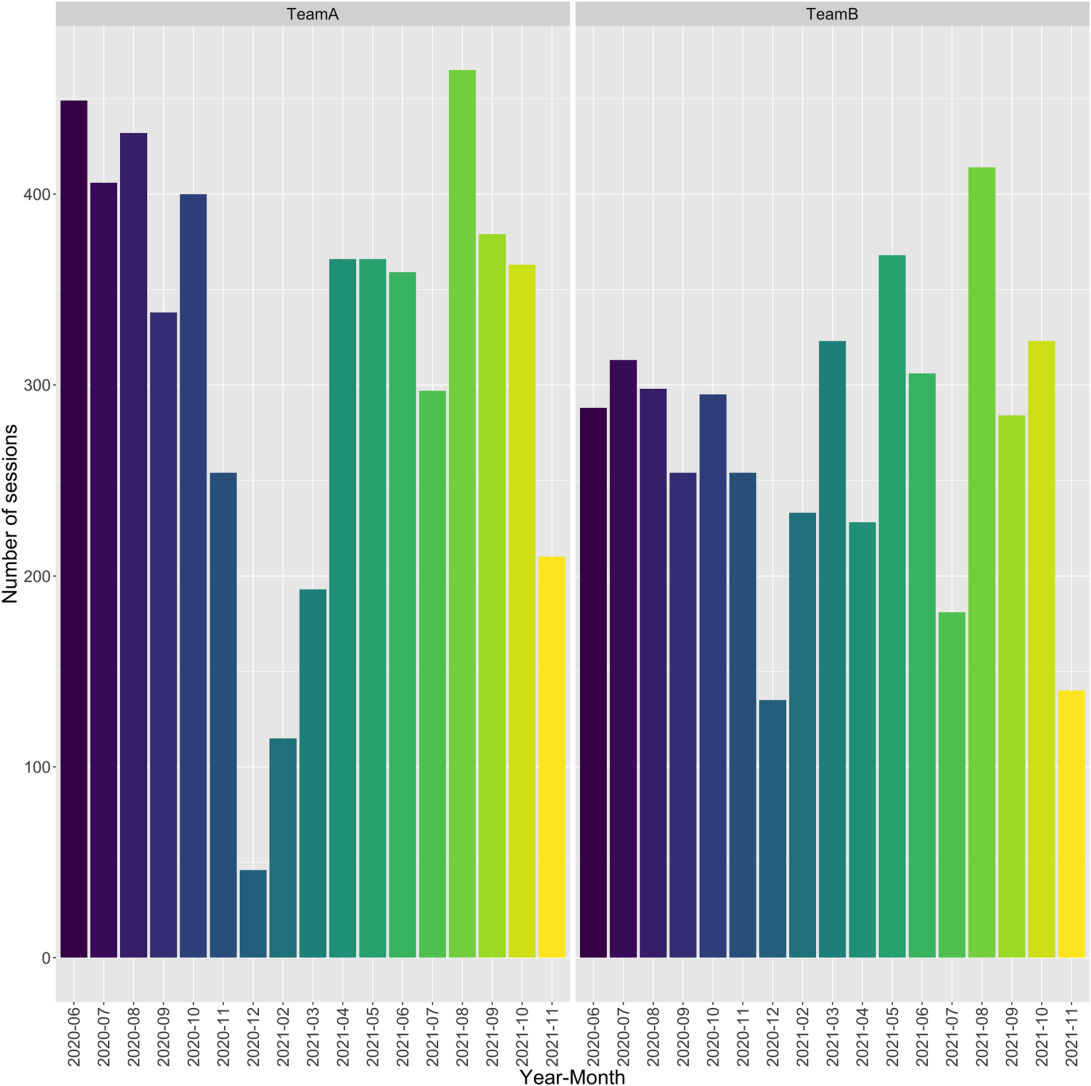
Number of GPS sessions per team, presented as a bar chart indicating team, year, and month.

The *SoccerMon* dataset contains 10,075 GPS reports in total, which are made up of 5,438 reports from Team A and 4,637 reports from Team B. Objective reports were collected from a total of 76 players, with 39 for Team A, and 37 for Team B. Figure 10 presents the number of GPS sessions (each yielding one GPS report) per player, and Fig. 11 presents the number of GPS sessions per month. Note that, due to the COVID-19 pandemic, the start of the 2020 season was pushed to June, while the 2021 season started in May.

**Fig. 12:**
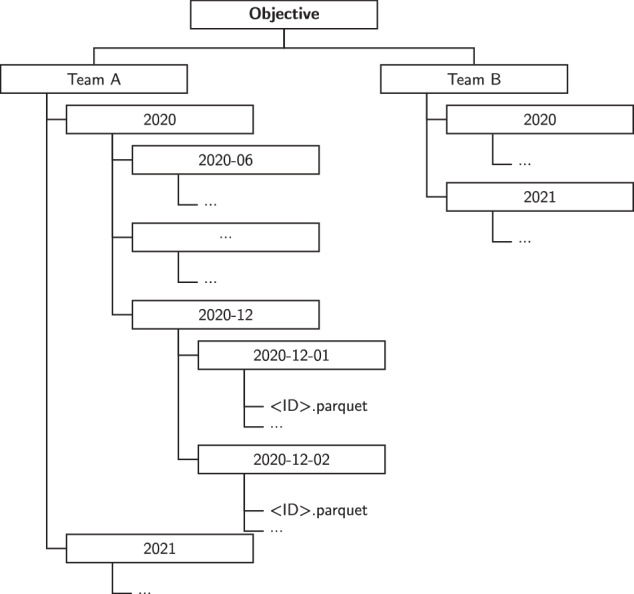
Folder structure for the second part of the *SoccerMon* dataset (objective). The files are hierarchically organized by team, year, month, and day.

The GPS reports are a collection of sensor measurements from a single unit captured within the duration of a session (period of time the unit was worn), and includes one entry (one row of data, or “record”) per measurement. Each record contains 17 parameters, including latitude, longitude, tri-axial acceleration, and rotation. Table 3 lists the GPS report parameters collected using the STATSports system. In the *SoccerMon* dataset, objective reports are organized hierarchically by team, year, month, and day. Figure 12 illustrates the folder structure for the second part of the *SoccerMon* dataset.

## Technical Validation

To validate and demonstrate the technical quality of the *SoccerMon* dataset, we performed multiple experiments, which provide certain baselines and provide insights into the statistical qualities of the dataset.

### Validation of subjective metrics

Validation of the subjective metrics was undertaken in three ways: initial self validation, correlation analysis, and parameter prediction, all serving as data quality indicators.

**Fig. 13:**
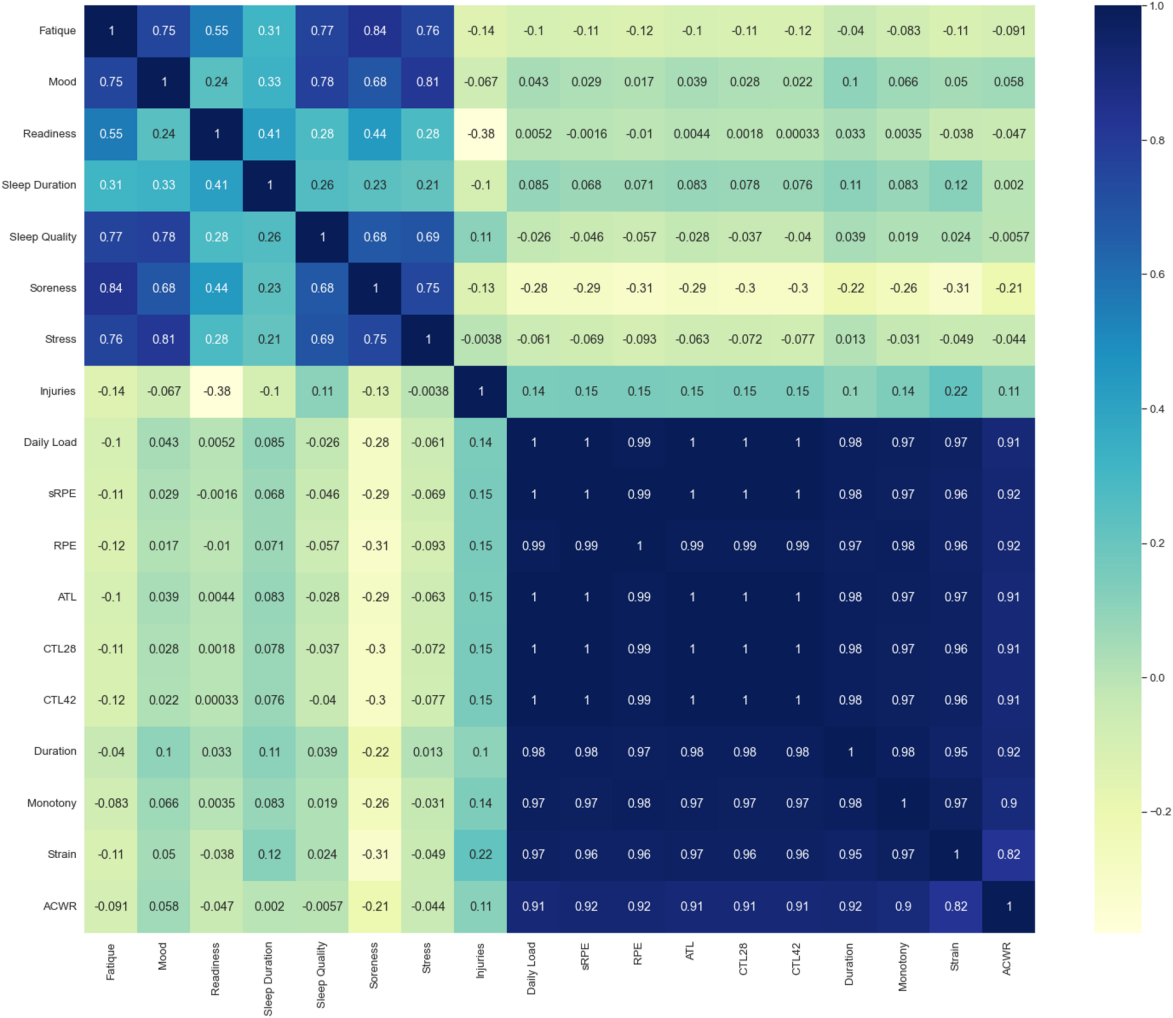
Correlation matrix of the self-reported subjective parameters in *SoccerMon*.
- **Parameter prediction:** We provide a small experiment where we perform a univariate prediction of the readiness-to-train parameter, following our work on predicting future values of the wellness parameters^27,28^. More details of such experiments using the *SoccerMon* dataset are given by Kulakou *et al*.^29^. As observed in Fig. 14, it is possible to predict the trends of such time-series data, i.e., enabling a more targeted planning of training sessions. In this particular experiment, we have trained an LSTMPlus machine learning model from the Tsai^36^ state-of-the-art Python deep learning library for time-series analysis, used a training split from the dataset (80%) to train the model, and used a test split from the dataset (20%) for testing. Building on previous knowledge^27,28^, we have trained the model on the entire team. Then, for unseen data from the test split, we used an input window of the 7 previous days to predict the following day. As observed in the figure, provided that appropriate hyper-parameters are found, the model can reasonably follow the trends in the data^29^. Moreover, even though predicting the upward and downward trends and catching peaks are more important than predicting the exact values, we looked at the Mean Squared Error (MSE), measuring the average sum of the square of the difference between the actual and the predicted values for all data points, i.e., $MSE=\frac{1}{n}{\sum }_{i=1}^{n}{\left({y}_{i}-{\widehat{y}}_{i}\right)}^{2}$. For the same parameter setting, the reported MSE is about 0.7 indicating that such predictions can be made with a high accuracy.

- **Self validation:** At the data collection stage, each participant was asked to verify their reported values before submitting their report to the *PmSys* system. Figures 1–5 present examples of this step, where the last screen of each questionnaire is a summary of the corresponding report to be validated by the participant.
- **Correlation analysis:** We performed a correlation analysis on the first part of the *SoccerMon* dataset. Figure 13 presents the correlation matrix of the self-reported subjective parameters in *SoccerMon*. The matrix presents the correlation coefficient for each combination of parameters. A perfect linear dependency is given when the correlation coefficient is one or minus one. No linear dependency is present when the correlation coefficient is zero. As can be observed from the figure, the correlation matrix appears to have three distinct blocks. The upper left block presents the intra-correlations among the wellness report parameters, the lower right block presents the intra-correlation among the training load report parameters, and the blocks in the lower left and upper right are the same, showing the inter-correlation between the wellness report parameters and the training load report parameters. The first noticeable observation of the correlation matrix is the high linear dependency among the training load parameters, as observed in the lower right part of the matrix. This dependency is due to the fact that all parameters pertaining to a training session are calculated from the parameters duration and RPE (see Table 2). The intra-correlations among the wellness parameters reveal valuable insights into the players’ well-being. Players’ stress is highly correlated to their mood, level of fatigue, and soreness. A lower stress level indicates lower fatigue and soreness, and vice versa. The highest correlation is found between soreness and fatigue. This relationship arises from the amount of training. The less sore a player is, the fresher she feels. This can also be seen by the negative correlation with daily load. A lower daily load indicates lower soreness. Readiness to train seems to correlate most with fatigue, soreness, and sleep duration, and we observe that sleep quality affects several parameters, whereas the session duration does not have the same effect. Further, sleep duration does not have as strong correlations as other relations. A lower correlation might indicate a strong circadian rhythm for elite soccer players and a structured sleep schedule. However, sleep quality is affected by other wellness parameters. The inter-correlations between wellness parameters and training load do not show as high correlations. Soreness is highest correlated with training parameters. Correlation measures a linear relationship between variables. Thus, a low correlation may suggest either the absence of a relationship or the presence of a non-linear association between the variables. These relations need a further investigation and advanced statistical modelling.

**Fig. 14:**
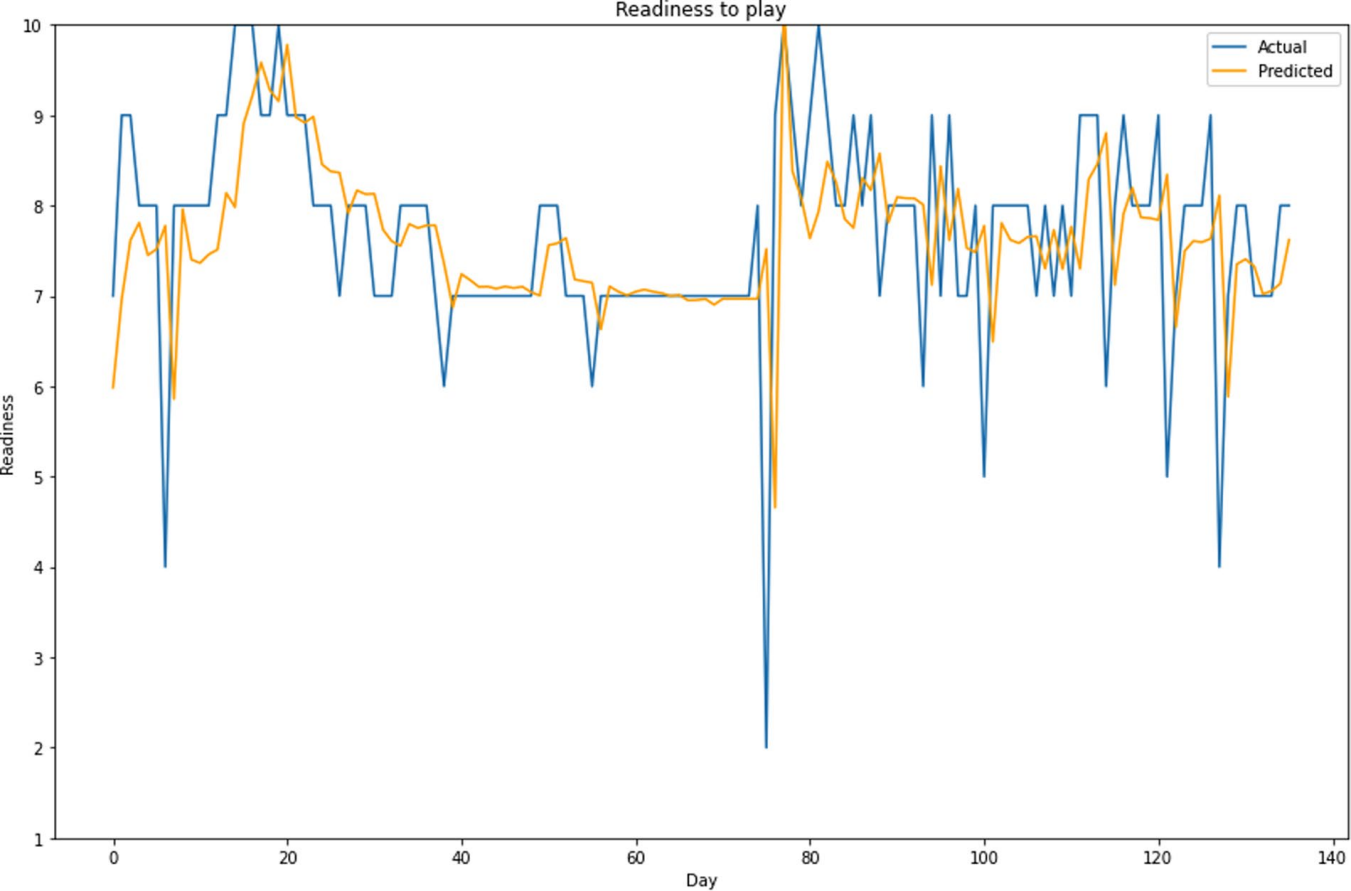
Actual and predicted values for the readiness-to-train parameter for a player from Team A. Training on entire team, number of epochs: 30, batch size: 5, input window: 7.

### Validation of objective metrics

Validation of the objective metrics was undertaken in three ways: GPS accuracy analysis, peak locomotor period analysis, and correlation analysis as data quality indicators.

**Fig. 15:**
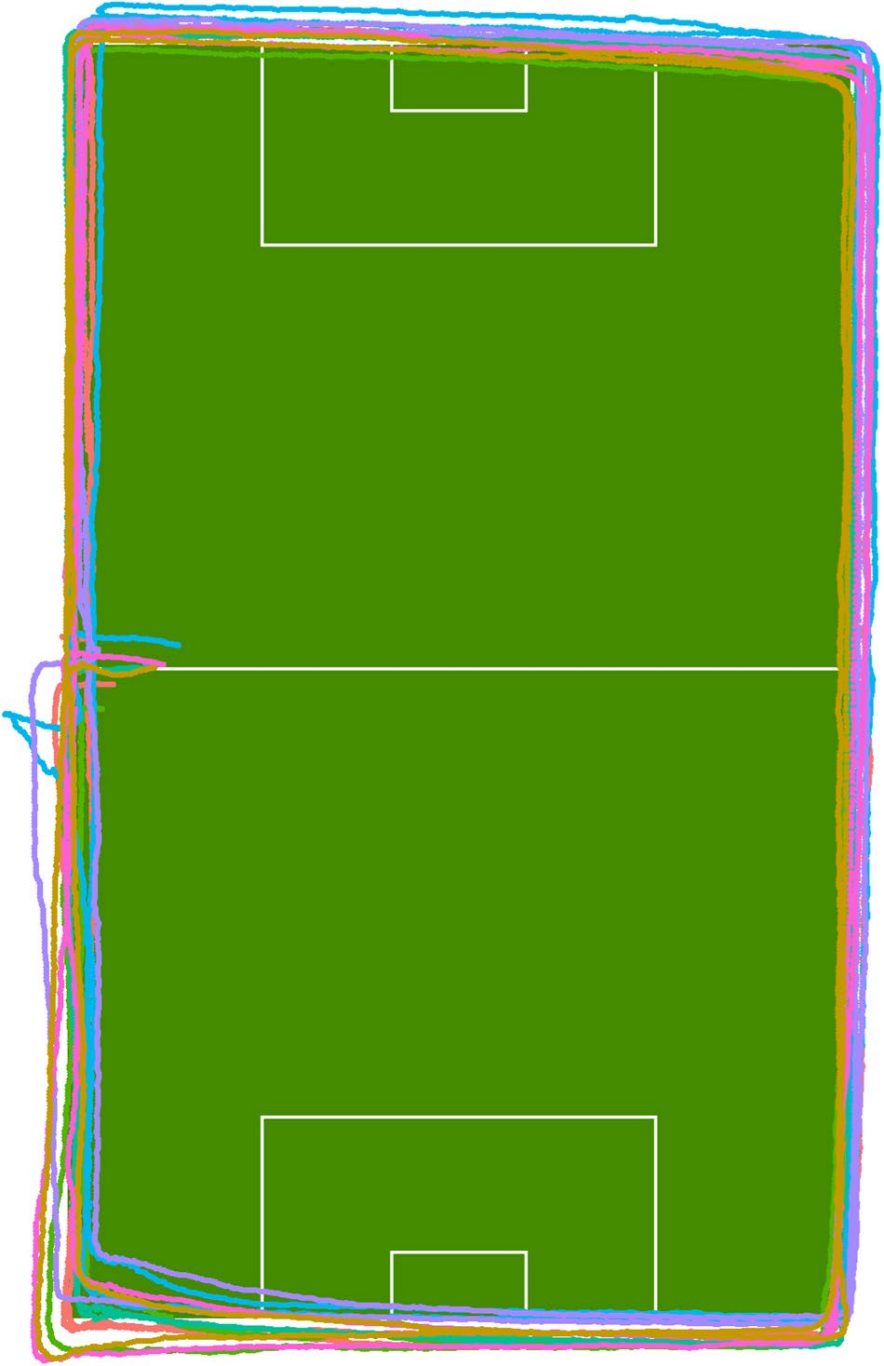
Measurement of the GPS accuracy for 7 players running on the lines around the pitch for two laps.
- **Correlation analysis:** Fig. 16 presents the correlation matrix of the objectively derived features of the tracked training sessions.

- **GPS accuracy:** To demonstrate the positional accuracy of the tracking data, we instructed seven players to jog clockwise around the pitch for two laps, following the side and end lines. As can be seen in Fig. 15, the player trajectories deviate slightly from the ground truth. In addition, there is also a slight player displacement from lap 1 to lap 2. A more thorough investigation of the validity and between-unit variability of STATSports Apex has previously been done by Beato *et al*.^6^. They reported a small distance bias of around 1–2% compared to the criterion distance of a 400-m athletic track, a specific team sports circuit of 128.5-m that replicated the movement demands of team sports (circuit performed on synthetic surface), and a 20-m trial (linear running). Peak speed showed a small bias of approximately 2% compared to the criterion measure (radar gun). They concluded that one can confidently use the system to measure distance and speed during training and match play.

**Fig. 16:**
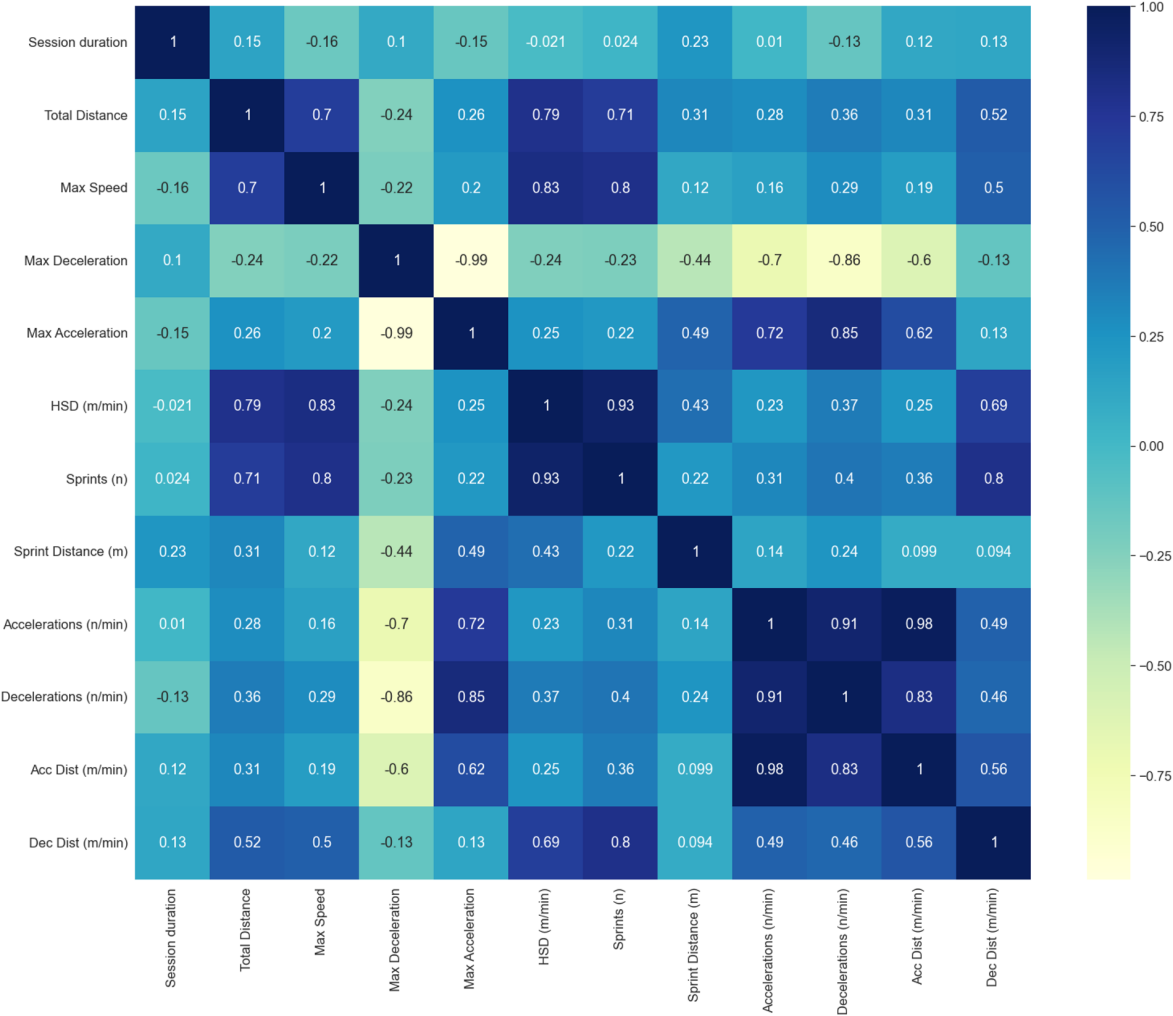
Correlation matrix of the features derived from objective parameters in *SoccerMon*.
- **Peak locomotor period analysis:** Soccer is characterized by its intermittent nature, with periods of high intensity followed by periods of low intensity, and so forth. Tracking data, to be considered valid, should be able to reflect this nature. Therefore, to further demonstrate the quality of our dataset, we showcase an experiment on the phenomenon of peak locomotor periods. Peak locomotor periods, also known as “high-intensity phases” or “worst-case scenarios”, are variable and duration-specific periods (usually 1, 5, or 10 minutes in length) within the session where, dependent on the type variable, the rolling sum or rolling average of that variable is the highest. Given the intermittent nature of soccer, one could hypothesize that the intensity during the subsequent period (often called the post-peak period) should be lower than that of the match average. To give an example, we reiterate the findings from Winther *et al*.^12^. In this study, we characterized the match-derived 1-minute peak locomotor periods for each playing position and compared them to the subsequent 5-minute period and the mean 5-minute period. To do this, we derived the following variables from a subset of the dataset: total distance (TD), high-speed running distance (HSRD) (>4.44 ms^−1^), sprint distance (SpD) (>5.55 ms^−1^), acceleration and deceleration distances (Acc_*dist*_ and Dec_*dist*_). Acc_*dist*_ and Dec_*dist*_ were defined as the distance covered with a positive or negative change in speed of more than ±2.26 ms^−2^, with a minimal effort duration of 0.3 s, finishing when the rate of acceleration/deceleration reached 0 ms^−2^. These thresholds were chosen according to previous research^37,38^. Finally, we used linear mixed modeling to estimate the differences between peak, next, and mean periods. As shown in Fig. 17, for most variables, the 1-minute peak period was significantly higher than both the subsequent 5-minute period and the mean 5-minute period. Furthermore, the distance covered during the subsequent 5-minute period was also significantly lower than that of the 5-minute match average, illustrating the transient reductions in running intensity following the peak periods. Taken together, these results show that our data reflect the intermittent nature of soccer^12^.

**Fig. 17:**
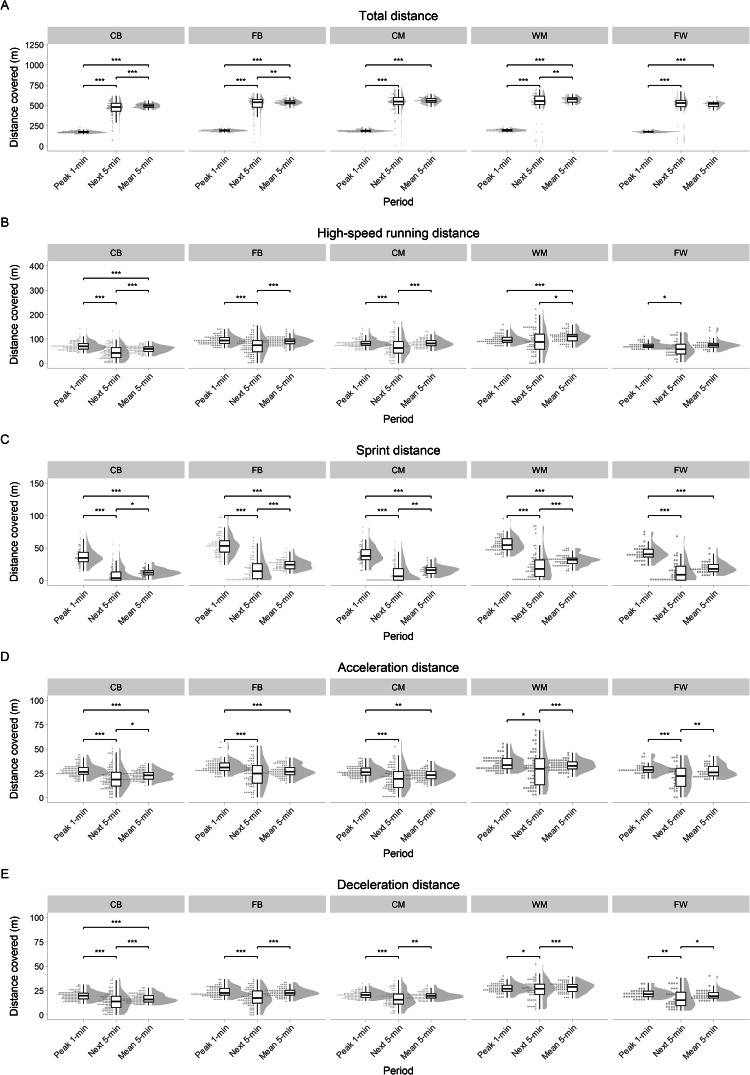
Distance covered during the peak 1-min, the next 5-min, and the mean 5-min period for: (**A**) total distance, (**B**) high-speed distance, (**C**) sprint distance, (**D**) acceleration distance, and (**E**) deceleration distance. Results are grouped by player position on the field: central defenders (CD), full-back (FB), central midfielder (CM), wide midfielder (WM), and forward (FW).

### Combined validation

**Fig. 18:**
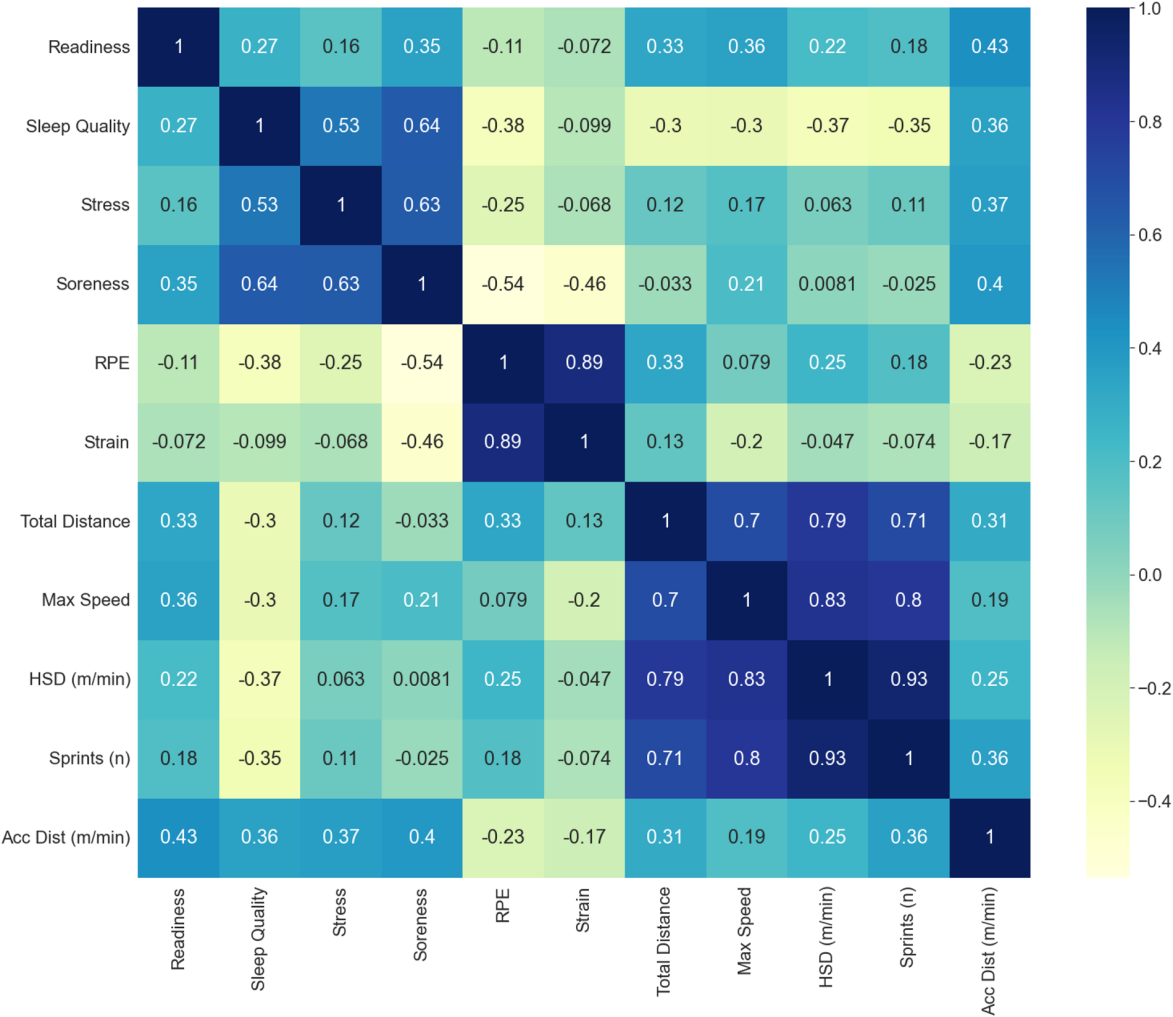
Correlation matrix of a subset of the combined subjective and objective parameters in *SoccerMon*.

The combination of subjective self-reports and objective tracking data gives a more holistic view of the soccer players and allows research on various applications. We analyze the correlation between the subjective and objective data to highlight the informative value. Due to a high number of features, we present the correlation matrix for a selected set of features. Figure 18 visualizes the correlation matrix of the subjective and objective features. We can observe that objectively measured acceleration distance reflects the subjective features of readiness, sleep quality, and stress. An increased acceleration distance increases readiness and improves sleep quality and stress levels. An interesting observation is the correlation of the perceived exertion sRPE and the GPS-based objective features such as acceleration distance, high-speed running distance (HSD)^39,40^, number of sprints and total distance. While the perceived exertion decreases with acceleration distance, it increases with the number of sprints, high-speed distance, and total distance. As correlation does not indicate causality, we can only speculate about the potential reasons for this behavior. An in-depth analysis is intended for future research on this dataset. An important first step is to address the issue of correlation dependence. Does the total distance impact the readiness of a player or vice versa? Additionally, other types of relationships than a linear one can be considered. The correlation matrix presented here is intended to highlight initial research questions that can be addressed based on this dataset.

## Usage Notes

In our research on analyzing the performance and wellness of soccer players, we collected, to the best of our knowledge, the largest and most diverse sports dataset consisting of both subjective and objective measurements over a period of two years from two different teams. In this paper, we make this dataset available as a resource to the research community, enabling researchers to study both sports and computer science aspects. We first outline some tools that are available in order to read, load, and use the data.

- **Data format for objective metrics:** Apache Parquet (https://parquet.apache.org) is a modern data storage format, which is beneficial for large columnar datasets. Parquet files can be accessed by any modern operating system. For example, MacOS supports *parquet-tools* which is a tool for processing parquet files using the command-line. *Apache Parquet Viewer* (https://github.com/mukunku/ParquetViewer) is a software solution for parquet files for *Microsoft*. Packages like *Apache Arrow* (https://arrow.apache.org) for the R, C++, Matlab, or Julia languages and *pandas*^41^ for the Python language provide convenient solutions to read, write, and manipulate parquet files.
- **Data loader:** A simple data loader which structures the subjective metrics in the dataset can be found in our GitHub repository: http://www.github.com/simula/soccermon.

*SoccerMon* can be used in many different ways, for sports and computer science. Here, we provide some examples of potential research areas.

- **Sports science:** *SoccerMon* enables researchers to study trends and developments in training load, wellness, and movement patterns during training and match play. The dataset also allows researchers to investigate how these parameters correlate and how they may influence each other. For example, one can look at differences between objective data and subjective reports, seasonal variations in training load, the impact of a match on sleep duration and quality the following days, contextual factors effect on physical match performance, the correlation between subjective sRPE and objective measured training load/intensity, and whether there are certain parameters, such as TD, HSRD, Sprint, and Acc, that have a stronger influence on subjectively experienced sRPE. It is also possible to use map overlays as a visualization tool for presenting and deriving insights from the GPS data. For further studies on GPS data analysis, readers are referred to the works of Winther *et al*.^12^, Pedersen *et al*.^16^, and Hoel^42^.
- **Injury analysis:** Injury prevention is a major field in sports research. The injury data provided in *SoccerMon*, albeit limited to subjective reports, can allow researchers to investigate the relationship between training load or wellness parameters and injury events. For instance, Jarmann^43^ analyzes injury risk based on different influence factors and applies survival analysis methods to predict injuries, while also discussing how to treat recurrent injuries (i.e., whether to consider each injury as a separate event, or to combine injuries that were reported on consecutive days into “injury periods”). Such research can allow stakeholders to understand injury risk better, and provide important insights about the steps to prevent injuries.
- **Time-series analysis:** *SoccerMon* is an example of a complex time-series dataset with many parameters and dimensions, and it can serve as an application scenario for various studies on time-series analysis, including AI/ML use cases. These range from predicting future values to finding better solutions for handling missing data (data imputation), possibly combining multiple parameters. For further studies on the prediction of subjective parameters, readers are referred to the works of Wiik *et al*.^27^, Johansen *et al*.^28^, Kulakou *et al*.^29^, and Sagbakken^44^.
- **Platform development:** The *PmSys* system was developed with the primary intention of replacing the traditionally manual (“pen and paper”) method of collecting information, still employed by many soccer teams worldwide, with a digital system for controlled systematic longitudinal data collection and extraction. It was established over multiple development cycles^28,31–33^, demonstrating the importance of continuous development of platforms with challenging system requirements and a diverse target audience (soccer players, coaches, medical personnel, and researchers). Potential future work can also focus on the design and implementation of such digital platforms, especially in terms of open source deployments, to serve as a tool for the scientific community.

Thus, *SoccerMon* enables a myriad of possibilities, not only within sports and computer sciences separately, but also at the intersection of the two areas as a more exciting opportunity. With this dataset, we encourage new contributions in the area, not only in terms of experiment reproducibility and method comparisons, but also in terms of the sharing of new datasets and tools with the community.

## Data Availability

We provide a sample codebase that can be used to import and structure the raw data from the *SoccerMon* dataset, as a public software repository on GitHub: http://www.github.com/simula/soccermon.
